# Neutrophil–Endothelium Interaction Mediated by S100A9 Promotes Pulmonary Vascular Remodeling During Pulmonary Hypertension

**DOI:** 10.1002/advs.202504397

**Published:** 2025-06-10

**Authors:** Yu Guo, Zhenqiang Gao, Ruoyang Zhang, Huanyu Long, Muzhi Zhang, Lin Liu, Zhe An, Yuezhe Shi, Ye Cui, Yufeng Jia, Lei Wang, Ying Sun, Jie Liu, Wei Wang

**Affiliations:** ^1^ Department of Immunology School of Basic Medical Sciences Capital Medical University Beijing 100069 China; ^2^ Department of Respiratory Medicine Capital Medical University Beijing 100069 China; ^3^ Immune Dysfunction and Pulmonary Fibrosis Joint Laboratory for Clinical Medicine Capital Medical University Beijing 100069 China; ^4^ Department of Respiratory and Critical Care Medicine Beijing Tiantan Hospital Capital Medical University Beijing 100070 China; ^5^ Department of Pulmonary and Critical Care Medicine Peking University Third Hospital Beijing 100191 China; ^6^ Department of Respiratory and Critical Care Medicine Beijing Chao‐Yang Hospital Capital Medical University Beijing 100020 China; ^7^ Department of Respiratory and Critical Care Medicine The Second Affiliated Hospital of Xi'an Jiaotong University Shaanxi 710004 China

**Keywords:** endothelial dysfunction, neutrophils, pulmonary vascular remodeling, S100A9

## Abstract

Pulmonary hypertension (PH) is a severe disease characterized by pulmonary vascular remodeling in which various immune cells play a critical role in vascular remodeling, although the details are still vague. Furthermore, current clinical treatments primarily focus on pulmonary vasodilation, but do not fundamentally address vascular remodeling itself. Here, first significant changes in neutrophils during the development of PH are demonstrated and show that neutrophil depletion can effectively attenuate disease progression. Moreover, the data show that neutrophil‐derived S100A9 is the key mediator to promote vascular remodeling, while both knockout and inhibition of S100A9 can prevent PH. In a co‐culture system of neutrophils and endothelial cells (ECs), hypoxic stimulation leads to increased S100A9 secretion by neutrophils, which activates the RAGE/PI3K/AKT pathway and causes dysfunction of ECs. These findings suggest that neutrophil‐derived S100A9 mediated neutrophil‐EC crosstalk plays an important role in pulmonary vascular remodeling, providing a promising strategy for treatment of PH.

## Graphical abstract

This study investigates the critical mechanisms underlying pulmonary hypertension progression, with a focus on the novel role of neutrophil‐derived S100A9 in endothelial dysfunction‐mediated pulmonary vascular remodeling. By integrating human lung samples, multi‐omics analyses, animal models, and mechanistic in vitro studies, it is revealed that how neutrophil–endothelial interactions mediated through the S100A9‐RAGE‐PI3K‐AKT signaling pathway contribute to endothelial dysfunction and vascular remodeling in PH.

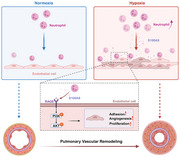

## 1 Introduction

Pulmonary hypertension (PH) is a heterogenous clinical disease characterized foremost by an abnormal increase in pulmonary artery pressure,^[^ ^1^ ^]^ universally defined by a mean pulmonary artery pressure > 20 mmHg.^[^ ^2^ ^]^ Across all its forms, pulmonary hypertension is associated with adverse vascular remodeling with obstruction and stiffening of the pulmonary vasculature.^[^ ^3^ ^]^ Without proactive management vascular remodeling leads to hypertrophy and ultimately failure of the right ventricle.^[^ ^4^ ^]^

The pathological mechanism underlying pulmonary vascular remodeling is intricate and remains incompletely understood. A key characteristic of vascular remodeling is the abnormal proliferation of structural cells and the accumulation of immune cells within and around the vascular wall,^[^ ^5^, ^6^ ^]^ in which the endothelium, as the innermost layer of the vascular wall, is at the forefront of sensing and responding to hemodynamic and hypoxic stimuli within blood vessels.^[^ ^7^ ^]^ Therefore, endothelial dysfunction is a common initiating event in many diseases characterized by vascular remodeling.

In recent years, immune and inflammatory responses in PH have gained significant attention because numerous studies have suggested that there is a close relationship between inflammation in the pathogenesis of PH.^[^ ^8^, ^9^, ^10^ ^]^ When immune cells are activated, they release large amounts of inflammatory factors, inducing endothelial cell proliferation and dysfunction, ultimately leading to vascular remodeling and right ventricular (RV) dysfunction.^[^ ^5^ ^]^ These findings suggest that implementing immunotherapy during PH progression may have a beneficial effect on the course of the disease. Therefore, it is urgent to clarify the inflammatory mechanisms during pulmonary vascular remodeling.

Our group have previously shown that various immune cells and inflammatory factors, including IL‐17, IL‐33, Th17 cells, and macrophages, are involved in endothelial dysfunction and vascular remodeling of PH.^[^ ^11^, ^12^, ^13^ ^]^ Furthermore, our analysis of transcriptomic and proteomic data showed that the common differentially expressed genes and proteins were primarily focused on inflammation‐ and immunity‐related molecules, in which S100A9 is one of the most significantly expressed molecules among them. S100A9, also known as calgranulin B or myeloid‐related protein14 (MRP‐14), is an endogenous damage‐associated molecular pattern molecule of the S100 calcium‐binding protein family.^[^ ^14^ ^]^ This molecule is mainly released by neutrophils,^[^ ^15^, ^16^ ^]^ and primarily functions as an inflammatory mediator, contributing to sustained inflammation and endothelial injury in septic or aged mice.^[^ ^17^, ^18^, ^19^ ^]^ Notably, S100A9 has been identified as a key regulator of inflammation, playing a critical role in the development and progression of various cardiovascular diseases—including atherosclerosis, myocardial infarction, aortic aneurysm, and peripheral arterial diseases, through modulating inflammatory responses, endothelial function, cellular proliferation, autophagy, apoptosis, and cell death.^[^ ^14^ ^]^ Up to date, however, the role of S100A9 in endothelial dysfunction‐mediated pulmonary vascular remodeling is still unclear.

In this context, we aimed to explore whether neutrophil‐derived S100A9 contribute to endothelial dysfunction in the development of PH and its potential mechanism, which hopefully provide novel therapeutic targets to mitigate pulmonary vascular remodeling and improve outcomes in patients suffering from PH.

## 2 Results

### 2.1 Increased Neutrophil Populations and Lung Infiltration in SuHx‐PH Mice

**Figure 1:**
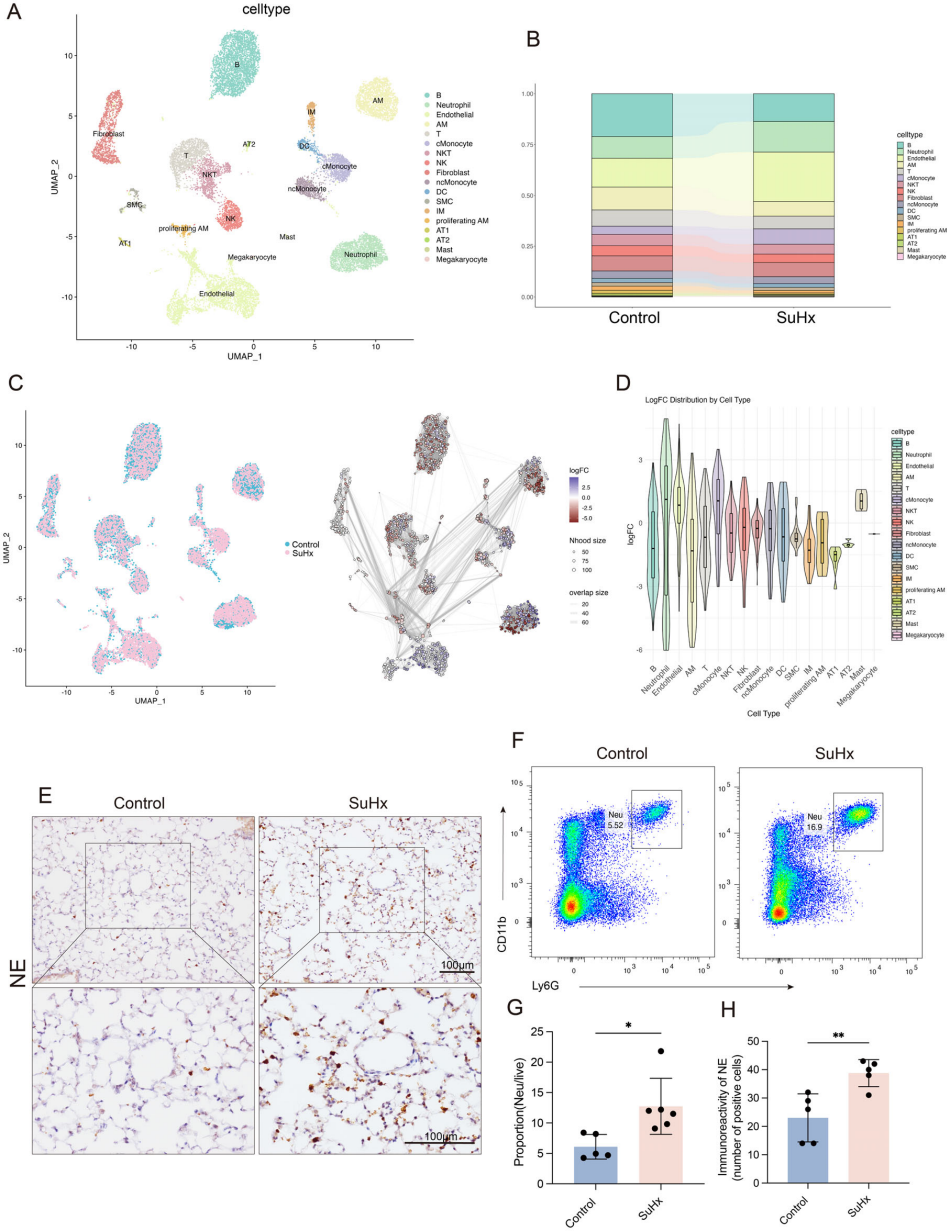
Increased neutrophil populations and lung infiltration in SuHx‐PH mice. A) UMAP visualization showed the presence of eighteen distinct lung cell types, highlighting the diversity of cell populations within the tissue. B) The percentage of neutrophils among total cells of mice with or without SuHx‐induced PH. The distribution of S100A9^+^ cells on the UMAP plot. C) UMAP showing cell neighborhood distributions from both groups (left), and differential abundance (DA) analysis using miloR, with neighborhoods colored by log fold change (SuHx‐PH vs.Control, right). Significant DA regions were identified by GLMM (FDR < 0.1). D) Violin plots show the distribution of log fold change (logFC) values for each annotated cell type. E) IHC for NE in lung sections of mice with or without SuHx‐induced PH (n = 5 per group). F) Representative flow cytometric images of CD11b^+^ Ly6G^+^ neutrophils among live cells in lung tissues of mice with or without SuHx‐induced PH. G) Flow cytometric analyses of lung tissues of mice (n = 5‐6 per group). H) Quantity analysis of NE‐positive cells. *p < 0.05, **p < 0.01. Unpaired t‐test was used.

To explore neutrophil dynamics in the context of PH, we constructed a SuHx‐induced PH mouse model and conducted scRNA‐seq on lung tissue. After sequencing, all cells were annotated based on their corresponding marker genes (Table S2, Supporting Information), identifying 18 distinct cell clusters (**Figure** **1**A). To assess cell population changes across experimental groups, we first performed clustering and cell type annotation based on the scRNA‐seq data (Figure 1B). A significant increase in the proportion of neutrophils was observed in the SuHx‐induced PH group compared to the control group. We further conducted differential abundance (DA) analysis using the miloR framework, as shown in Figure 1C. The left panel displays uniform manifold approximation and projection (UMAP) projections of Control group (blue) and SuHx‐induced PH group (pink) cells, while the right panel shows DA results highlighting differential cell abundance across conditions. Figure 1D illustrates a honeycomb plot based on log2 fold change, which further confirms a substantial enrichment of neutrophil populations in the SuHx‐induced PH group, suggesting a potential involvement of these cells in disease progression. To further validate the increased neutrophil infiltration observed in the lungs of SuHx‐induced PH mice, immunohistochemical staining for neutrophil elastase (NE) was conducted. The results demonstrated a significant elevation in NE‐positive cells within the pulmonary tissues of SuHx‐induced PH mice, indicating enhanced neutrophilic activity (Figure 1E). Complementary flow cytometry analysis of CD11b^+^Ly6G^+^ neutrophil populations confirmed a marked increase in neutrophil abundance in the SuHx‐induced PH group relative to controls (Figure 1F).

Taken together, these results suggest a potential role of neutrophils in the pathogenesis of PH.

### 2.2 Neutrophil Depletion Attenuates Symptoms of Mice with SuHx‐Induced PH

**Figure 2:**
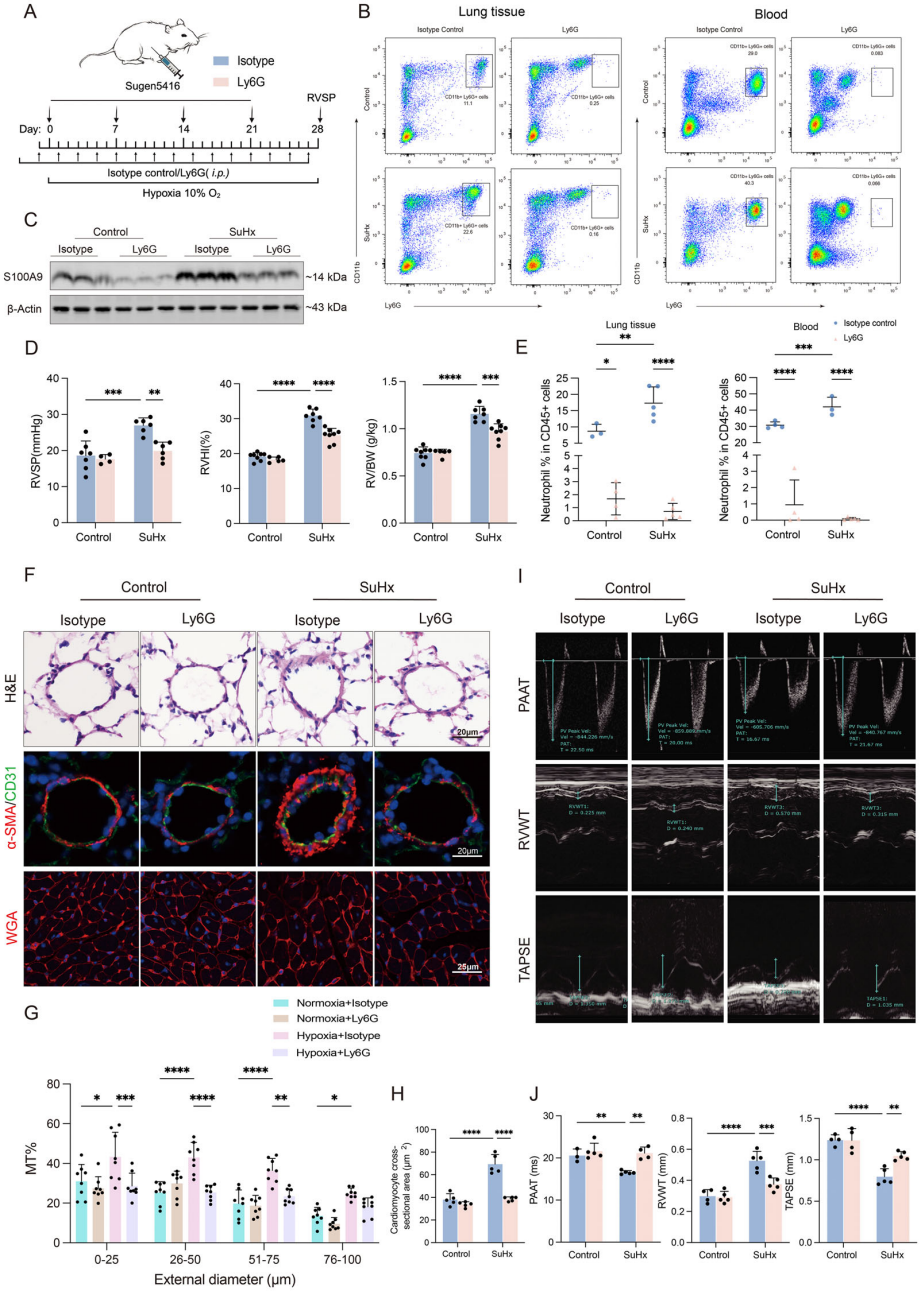
Neutrophil deficiency attenuates PH in mice. A‐J: Anti‐Ly6G antibody and isotype (i.p.) were administered to C57BL/6 mice under control and SuHx‐induced conditions. A) Schematic diagram for establishing experimental murine model. B) Representative flow cytometric images of CD11b^+^ Ly6G^+^ neutrophils among CD45^+^ cells in lung tissues (left panel) and blood (right panel) of experimental mice. C) Representative Western blot of S100A9 in the lungs of experimental mice. D) RVSP, RVHI and RV/BW of mice (n = 4‐8 per group). E) Flow cytometric analyses of lung tissues (left panel) and blood (right panel) of mice (n = 3‐5 per group). F) Representative images of H&E and IF staining for α‐SMA/CD31 in lung sections of experimental mice, as well as representative images of WGA staining showing cardiomyocyte hypertrophy in the RV. G) Quantification of wall thickness of the pulmonary vasculature for vessels of 0–25 µm, 26–50 µm, 51–75 µm and 76–100 µm in diameter (n = 8 per group). H) Quantification of WGA staining (n = 5 per group). I) Echocardiography showing the representative figures of PAAT, RVWT and TAPSE. J) Statistical analysis of PAAT, RVWT and TAPSE (n = 3‐5 per group). *p < 0.05, **p < 0.01, ***p < 0.001, ****p < 0.0001. Two‐way ANOVA was performed, followed by Bonferroni correction for six pairwise comparisons among the four experimental groups.

Because studies derived from ours and others have shown that there are increases in neutrophil numbers which might associate with disease progression in SuHx‐induced PH, we further investigated the role of neutrophil in the pulmonary vascular remodeling. To achieve this, an antibody against Ly6G was employed. It has been known that Ly6G is a glycoprotein specifically expressed on the surface of neutrophils, and antibodies targeting Ly6G results in immune‐mediated neutrophil depletion.^[^ ^20^, ^21^ ^]^ To clarify role of neutrophils in SuHx‐induced PH pathogenesis, we depleted neutrophils in mice by administering intraperitoneal injections of an anti‐Ly6G antibody (200 µg on alternate days) and established a SuHx‐induced PH model in both anti‐Ly6G‐treated and isotype control‐treated mice (**Figure** **2**A). The results showed that there were significantly reduced percentages of neutrophils in the lung tissues and blood of anti‐Ly6G‐treated mice compared to those of isotype control‐treated mice (Figure 2B,E; Figure S2A,B, Supporting Information). After neutrophil depletion, a population of CD11b^+^ and Ly6G^low^ cells still remained, which is possibly due to the stimulation of neutrophil precursors in the bone marrow, leading to an increase in newly generated neutrophils.^[^ ^21^ ^]^ However, these cells are not capable of performing their full functional roles. Western blot confirmed that S100A9 expression was also markedly reduced in the lung tissues of the anti‐Ly6G‐treated group (Figure 2C). Additionally, anti‐Ly6G‐meidiated neutrophil depletion resulted in lower levels of RVSP, right ventricular hypertrophy index (RVHI), and RV weight radio (right ventricular/body weight (RV/BW) of mice with SuHx‐induced PH (Figure 2D). Since vascular remodeling in PH primarily occurs in the distal pulmonary arterioles, we calculated percent medial thickness in intrapulmonary arterioles with a diameter of <100 µm. Consistent with these results, we observed the significantly reduced vascular remodeling <100 µm (Figure 2F,G), and cardiomyocyte hypertrophy (Figure 2F,H) in neutrophil‐depleted SuHx‐induced PH mice. Pulmonary vascular remodeling leads to increase pulmonary arterial pressure, RV hypertrophy and RV dysfunction, which is a hallmark of end‐stage PH^3^. Furthermore, RVWT was significantly reduced after anti‐Ly6G treatment, while analysis of RV function, including PAAT and TAPSE, showed that there was a marked improvement (Figure 2I,J).

### 2.3 S100A9 Expression is Upregulated in the Lungs and Predominantly Originates from Neutrophils during the Development of PH

**Figure 3:**
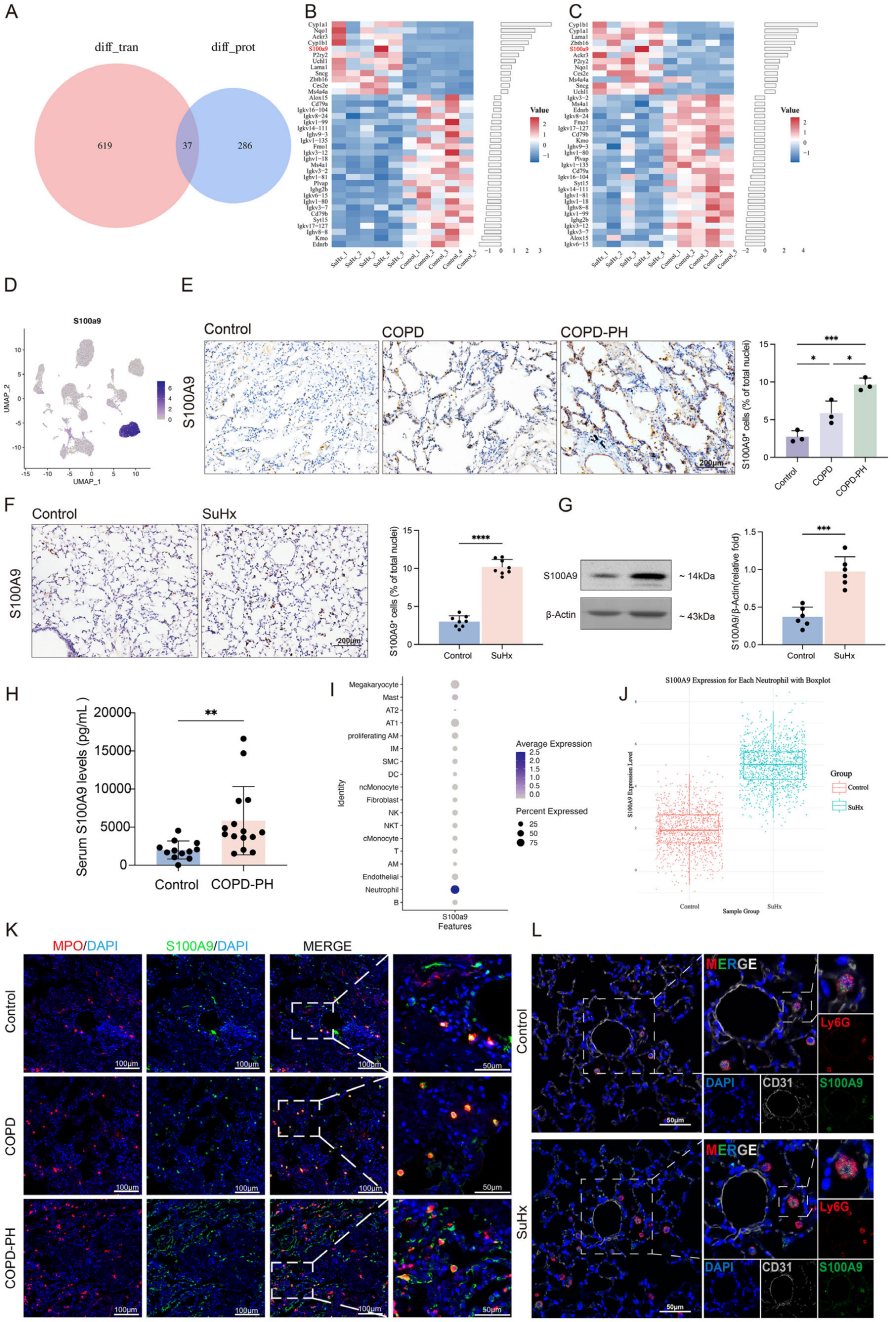
S100A9 is upregulated in the lungs during the pathogenesis of PH. A) Venn diagram showing the overlap between DEGs from transcriptomic analysis and DEPs from proteomic analysis. B) Heatmap of the 37 differentially genes identified in the SuHx‐induce PH model (|log_2_FC| ≥ 1.5, FDR < 0.05). C) Heatmap of the 37 differentially expressed proteins identified in the SuHx‐induce PH model (|log_2_FC| ≥ 1.0, FDR < 0.05). D) UMAP plot visualizing the expression of *S100A9* across all cells. Color intensity indicates the normalized expression level of the gene. E) IHC and quantity analysis of S100A9 in sections of human lung tissues (3 groups, n = 3 per group). F) IHC and quantity analysis of S100A9 in sections of the lung tissues of SuHx‐induced PH mice (n = 8 per group). G) Western blot analysis of S100A9 in the lung tissues of the experimental mice (fold change of SuHx versus Control group, n = 6 per group). H) ELISA analysis of serum levels of S100A9 in patients with COPD‐PH and control groups (n = 12‐15 per group). I) Dot plot showing the expression level of S100A9 across different cell types. J) Boxplot showing the expression level of S100A9 in neutrophils across different groups. K) Representative images of IF staining for MPO (red) and S100A9 (green) in sections of the lung tissues of healthy controls, COPD patients without PH and COPD‐PH patients. Nuclei were counterstained with DAPI (blue). L) Representative images of IF staining for CD31 (white), Ly6G (red) and S100A9 (green) in sections of the lung tissues of mice with or without SuHx‐induced PH. Nuclei were counterstained with DAPI (blue). Data are presented as mean ± SD deviation, normalized to β‐actin relative gene expression was calculated using the 2^(‐ΔΔCt) method. *p < 0.05, **p < 0.01, ***p < 0.001, ****p < 0.0001. One‐way ANOVA was performed, followed by Bonferroni post hoc correction for three pairwise comparisons among the three experimental groups (E). Unpaired *t*‐test was used for comparisons in panels F‐H.

To further investigate the specific molecules and underlying mechanisms by which neutrophils exert their effects in PH, we conducted integrative multi‐omics analyses. Transcriptomic and proteomic profiles were obtained from the lung tissues of SuHx‐induced PH mice. Transcriptome sequencing identified 656 differentially expressed genes (DEGs), while proteomics analysis revealed 323 differentially expressed proteins (DEPs, **Figure** **3**A). Cross‐analysis showed 37 overlapping differentially expressed genes and proteins (Figure 3A), among which S100A9, a protein involved in inflammatory events,^[^ ^22^ ^]^ was significantly upregulated (Figure 3B,C). It has been shown that S100A9 is predominantly derived from monocytes and neutrophils in vivo, accounting for ≈40% of neutrophil cytosolic proteins, and functions as a key alarmin‐like molecule.^[^ ^23^ ^]^ We further validated the significant upregulation of S100A9 in scRNA‐seq data from the same mouse model, confirming its elevated expression (Figure 3D). As shown in Figure 3E, immunoreactivity for S100A9 was significantly higher in lung sections of COPD patients with PH than those of controls and COPD patients without PH. Correspondingly, data of immunostaining and Western blot showed that the protein expression of S100A9 significantly increased in the lung tissues of SuHx‐induced PH mice (Figure 3F,G), chronic hypoxia‐induced PH mice and rats with MCT‐induced PAH (Figure S3A,B, Supporting Information) compared to those of control animals. Additionally, ELISA analysis showed that serum levels of S100A9 were significantly elevated in patients with the COPD‐PH compared to those of controls (Figure 3H). We further analyzed the expression profile of S100A9 using scRNA‐seq data, revealing that S100A9 was predominantly expressed in neutrophils (Figure 3I), with significantly higher expression levels observed in neutrophils of SuHx‐induced PH mice (Figure 3J). This finding was further supported by immunofluorescence staining in which the number of neutrophils was markedly increased in the lung tissues of both COPD and COPD‐PH patients compared with those of controls, and the most S100A9 immunoreactivities were co‐localized with neutrophils (Figure 3K). Similar patterns were observed in the lung tissues of SuHx‐PH mice, where an increased number of S100A9‐positive neutrophils were found surrounding pulmonary endothelial cells (Figure 3L).

### 2.4 S100A9 Deficiency Attenuates Symptoms of Mice with SuHx‐Induced PH

**Figure 4:**
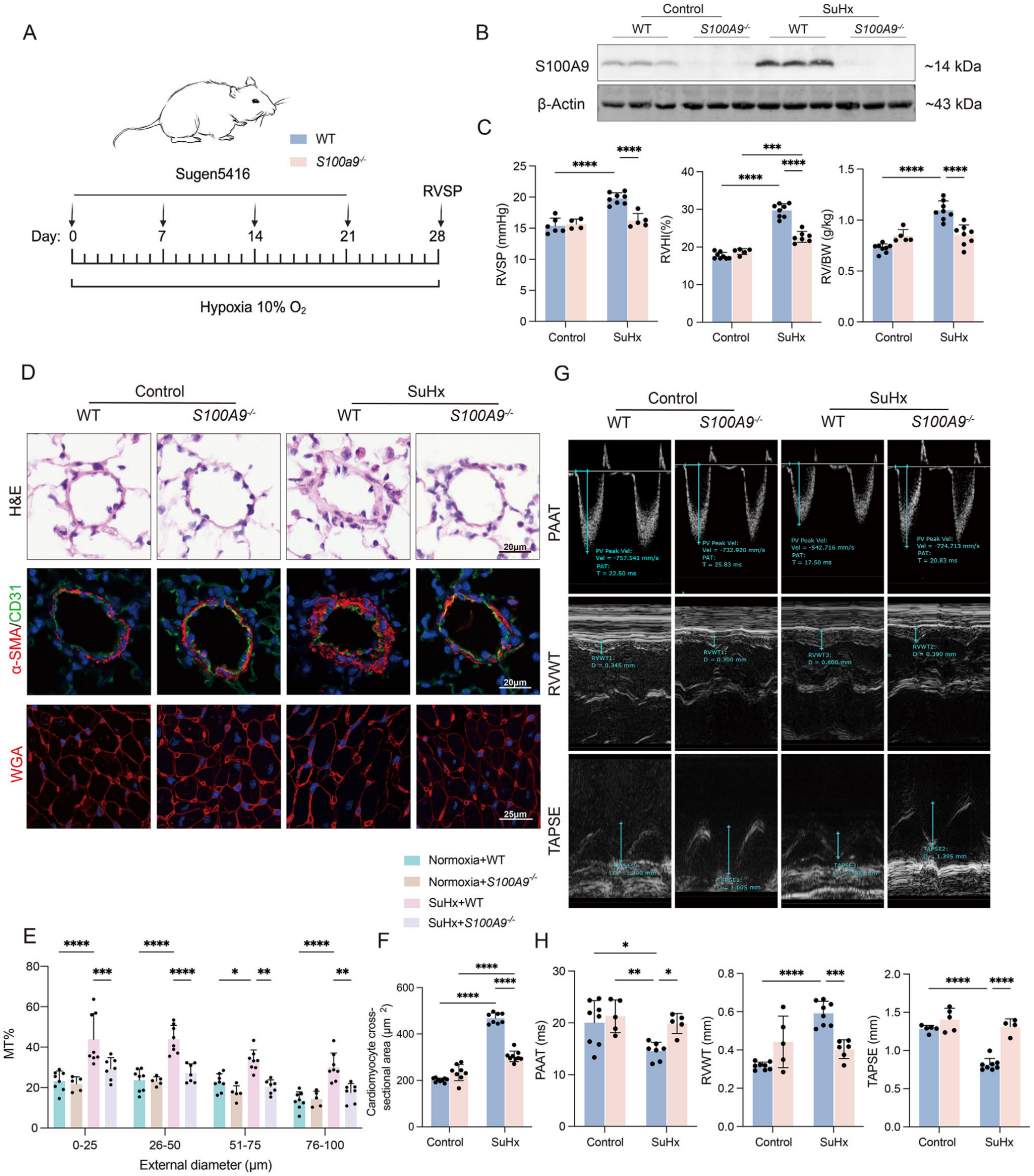
S100A9 deficiency attenuates symptoms of mice with SuHx‐induced PH. A) Schematic diagram of establishment of experimental murine model. B‐H: WT or *S100A9^−/−^* mice were under control and SuHx‐induced conditions. B) Representative Western blot of S100A9 in the lungs of experimental mice. C) RVSP, RVHI and RV/BW of mice (n = 4‐8 per group). D) Representative images of haematoxylin and eosin (H&E) and α‐SMA (smooth muscle α‐actin)/CD31 IF staining in lung sections of the experimental mice, as well as representative images of WGA staining showing cardiomyocyte hypertrophy in the RV. E) Quantification of wall thickness of the pulmonary vasculature of the experimental mice (n = 5‐8 per group). F) Quantification of WGA staining (n = 8‐9 per group). G) Echocardiography showing representative images of PAAT, RVWT and TAPSE. H) Statistical analysis of PAAT, RVWT and TAPSE (n = 4‐8 per group). *p < 0.05, **p < 0.01, ***p < 0.001, ****p < 0.0001. Two‐way ANOVA was performed, followed by Bonferroni correction for six pairwise comparisons among the four experimental groups.

To investigate whether change in S100A9 expression influences the phenotype in PH development, S100A9 knockout (*S100A9^−/−^*) mice were used to establish a SuHx‐induced PH model (**Figure** **4**A). Western blot showed that there was the absence of S100A9 protein in the lung tissues of the *S100A9^−/−^* group (Figure 4B). Data also showed that the absence of S100A9 significantly attenuated the increase in RVSP, reduced the extent of RVHI and RV/BW (Figure 4C) in mice with SuHx‐induced PH. The results showed that treatment with SuHx induced significant remodeling of these arterioles in WT mice, while *S100A9^−/−^* mice exhibited reduced remodeling (Figure 4D,E). WGA staining (Figure 4D,F) and echocardiography in mice revealed that *S100A9^−/−^* significantly mitigated SuHx‐induced RVWT and cardiomyocyte hypertrophy, and improved RV function, including PAAT and TAPSE (Figure 4G,H).

### 2.5 The S100A9 Inhibitor Paquinimod Attenuates Symptoms of Mice with SuHx‐Induced PH

**Figure 5:**
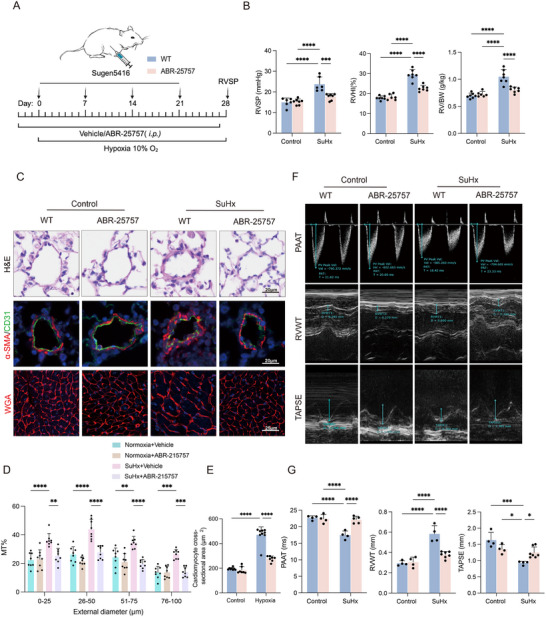
The S100A9 inhibitor Paquinimod attenuates symptoms of mice with SuHx‐induced PH. A) Schematic diagram of the establishment of experimental murine model. B‐H: ABR‐25757(i.p.) was administered to C57BL/6 mice under control and SuHx‐induced conditions. B) RVSP, RVHI and RV/BW of mice (n = 6‐7 per group). C) Representative images of H&E and α‐SMA/CD31 IF staining in lung sections of the experimental mice, as well as representative images of WGA staining showing cardiomyocyte hypertrophy in the RV. D) Quantification of wall thickness of the pulmonary vasculature (n = 8 per group). E) Quantification of WGA staining (n = 7‐9 per group). F) Echocardiography showing the representative images of PAAT, RVWT and TAPSE. G) Statistical analysis of PAAT, RVWT and TAPSE, per group (n = 4‐7 per group). *p < 0.05, **p < 0.01, ***p < 0.001, ****p < 0.0001. Two‐way ANOVA was performed, followed by Bonferroni correction for six pairwise comparisons among the four experimental groups.

To further clarify the role of S100A9 in mice with SuHx‐induced PH, an S100A9 inhibitor Paquinimod (ABR‐25757) was used to examine its effects on disease progression. ABR‐25757, a small‐molecule drug, has primarily been studied for its potential immunomodulatory effects, one of which is S100A9 inhibition effect.^[^ ^24^ ^]^ The inhibitor ABR‐25757 was first given three days before establishing the SuHx‐induced PH model and continuatively given daily via intraperitoneal injection (10 mg·kg^−1^/per dose) throughout the experiment^[^ ^25^, ^26^ ^]^ (**Figure** **5**A). Results showed that administration of ABR‐25757 effectively reduced SuHx‐induced increases in RVSP, RVHI and RV/BW (Figure 5B) compared to those of controls. Immunostaining indicated that vascular thickening was less pronounced in sections of the lung tissues (Figure 5C), while cardiomyocytes hypertrophy of SuHx‐induced PH mice treated with ABR‐25757 was also improved (Figure 5D,E). Additionally, ABR‐25757 treatment also significantly reduced RV wall thickness, with marked improvement in RV function parameters, including PAAT and TAPSE (Figure 5F,G).

### 2.6 Hypoxia‐Induced Neutrophil‐Endothelial Interaction Promotes Endothelial Dysfunction via S100A9‐Mediated Activation of the RAGE‐PI3K‐AKT Pathway

**Figure 6:**
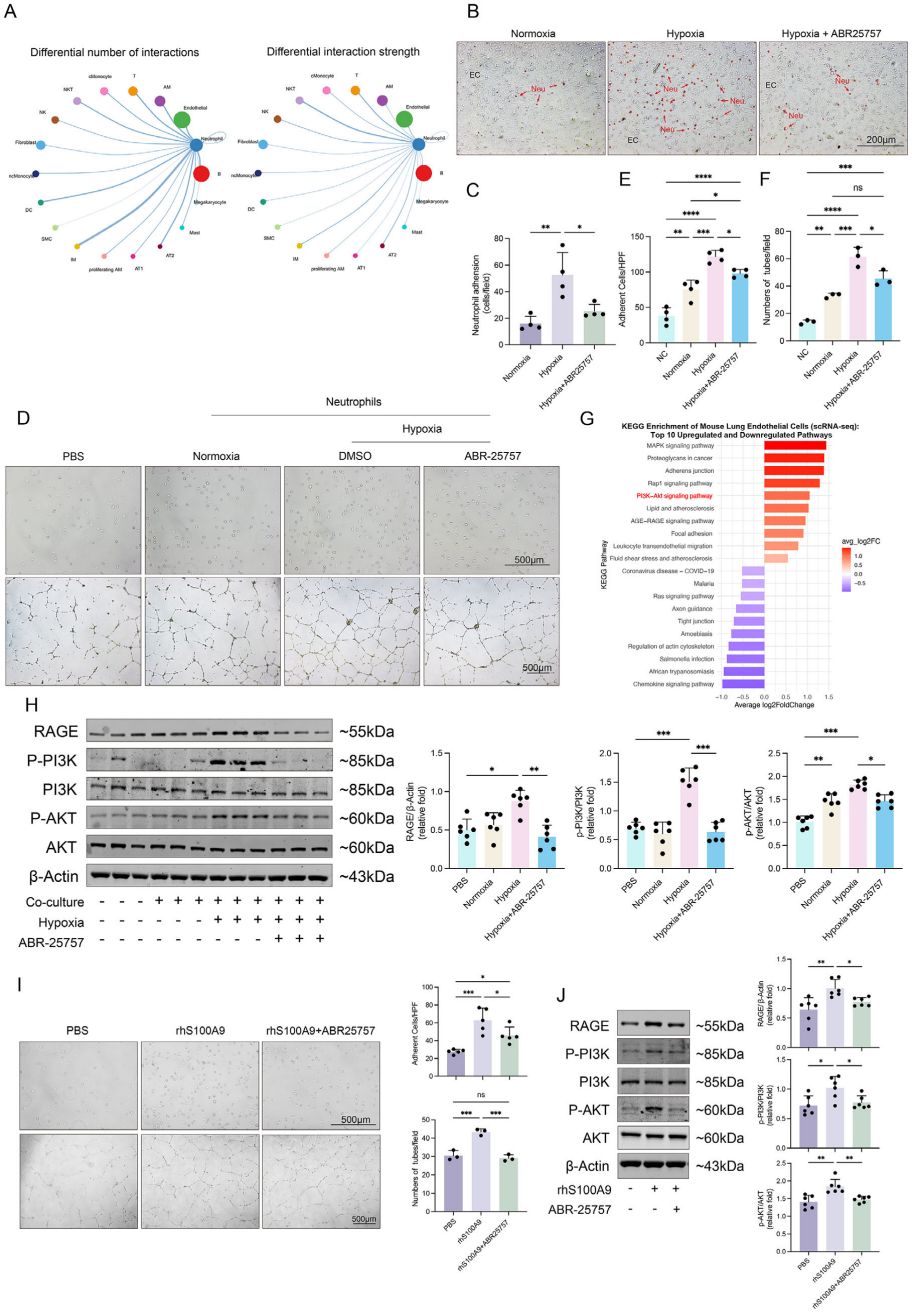
Hypoxia‐induced neutrophil‐endothelial interaction promotes endothelial dysfunction via S100A9‐mediated activation of the RAGE‐PI3K‐AKT pathway. A) Number and strength of cellular interactions were analyzed using ‘CellChat’, with results shown in a circle plot. B) Representative images of the neutrophil adhesive assay. The red arrows point at the PKH26‐labelled neutrophil (red). C) Quantification of the neutrophil adhesive assay (n = 4 per group). D) Representative images of adhesion and angiogenesis of hPAESs co‐cultured with neutrophil under hypoxic or normoxic conditions, in the presence or the absence of ABR‐25757. E) Quantification of adhesive hPAECs (n = 4 per group). F) Quantification of angiogenesis of hPAECs (n = 3 per group). G) KEGG pathway enrichment analysis of mouse lung endothelial cells. H) Representative Western blot of hPAECs co‐cultured with neutrophils under normoxic or hypoxic conditions, in the presence or the absence of ABR‐25757, showing protein expression of the RAGE/PI3K/AKT pathways in hPAECs (n = 6 per group). I) Representative images and quantification of adhesion and angiogenesis of hPAECs evaluated after stimulation with rhS100A9 or treated with ABR‐25757 (n = 3‐5 per group). J) Representative Western blot and quantification of hPAECs induced by rhS00A9 or treated with ABR‐25757, focusing on the RAGE/PI3K/AKT pathways (n = 6 per group). ^ns^ *p* > 0.05, *p < 0.05, **p < 0.01, ***p < 0.001, ****p < 0.0001. One‐way ANOVA was performed, followed by Bonferroni post hoc correction for three pairwise comparisons among the three groups (C, I, and J) and the six pairwise comparisons among the four experimental groups (E, F, and H).

As we demonstrated that neutrophils and the secreted S100A9 play a critical role in PH progression, while neutrophils primarily localized around CD31^+^ ECs in lung tissue. To further investigate which structural cells interacted more closely with neutrophils in the lungs, we used the ‘CellChat’ R package to analyze differential interactions number across all cell types. The data showed that from an interaction strength perspective, neutrophils seemed to have potential effects on ECs, and the number of interactions between neutrophils and ECs also significantly increased (**Figure** **6**A; Figure S4A, Supporting Information). S100A9 primarily signals through its receptors RAGE and TLR4. To investigate the expression patterns of these receptors, we first analyzed scRNA‐seq data from the lung tissues. TLR4 expression was undetectable at the transcriptomic level. Furthermore, protein‐level analysis following exogenous stimulation with S100A9 showed no significant induction of TLR4 expression in endothelial cells (Figure S4B, Supporting Information). In contrast, RAGE (encoded by the Ager gene) was found to be more highly expressed in endothelial cells than that of smooth muscle cells (Figure S4C, Supporting Information). Notably, Ager expression was significantly upregulated in endothelial cells of the SuHx‐induced PH mice compared to those of the control mice (Figure S4D, Supporting Information). These findings suggest that RAGE, rather than TLR4, may serve as the predominant receptor mediating S100A9 signaling in endothelial cells during the PH pathogenesis.

Using a neutrophil adhesion assay, we found that hypoxic conditions increased neutrophil adhesion to ECs, which was reduced by S100A9 inhibitor ABR‐25757 (Figure 6B,C). To test the hypothesis that hypoxia enhances neutrophil‐endothelial cell interactions and affects endothelial function, we co‐cultured neutrophils isolated from healthy human peripheral blood with hPAECs. The results showed a marked increase in endothelial adhesion and angiogenic capacity when co‐cultured with neutrophils in a transwell system under either hypoxic condition (Figure 6D–F) or stimulated with supernatants collected from hypoxia‐exposed neutrophils (Figure S4F, Supporting Information), all of which, again, were attenuated by ABR‐25757.

Next, to further investigate the mechanisms underlying S100A9‐mediated endothelial dysfunction, we extracted endothelial cell populations from the scRNA‐seq dataset and conducted KEGG and GSEA pathway enrichment analyses. The results revealed significant alterations in the PI3K‐AKT and AGE‐RAGE signaling pathways (Figure 6G; Figure S4E, Supporting Information), in which the RAGE‐PI3K‐AKT pathway was activated in ECs, while ABR‐25757 significantly attenuated these effects (Figure 6H; Figure S4G, Supporting Information).

CCK8 assay showed that treated hPAECs with recombinant human S100A9 protein (rhS100A9, 10 µg mL^−1^) significantly promoted cell proliferation (Figure S4H, Supporting Information) Following this assay, the concentration of 10 µg mL^−1^ was used in subsequent experiments, showing that rhS100A9 significantly enhanced endothelial adhesion and angiogenesis (Figure 6I), along with activation of the corresponding signaling pathways. Again, these effects were reversed by adding the inhibitor (Figure 6J).

To further confirm the critical role of the RAGE–PI3K–AKT pathways in mediating endothelial dysfunction under high S100A9 conditions, we replaced the S100A9 inhibitor ABR‐25757 with a PI3K inhibitor (LY294002) and repeated the aforementioned endothelial functional assays. The results demonstrated that LY294002 also effectively ameliorated endothelial dysfunction induced by elevated S100A9 levels or neutrophil exposure (Figure S5A–C, Supporting Information).

Taken together, these data suggest that hypoxia enhances neutrophil‐endothelial cell interactions, leading to endothelial dysfunction and subsequent vascular remodeling, in which neutrophil‐derived S100A9 is likely involved though activating the RAGE‐PI3K‐AKT signaling pathways of ECs.

### 2.7 S100A9‐RAGE‐PI3K‐AKT Pathways Participate in the Process of SuHx‐Induced PH

**Figure 7:**
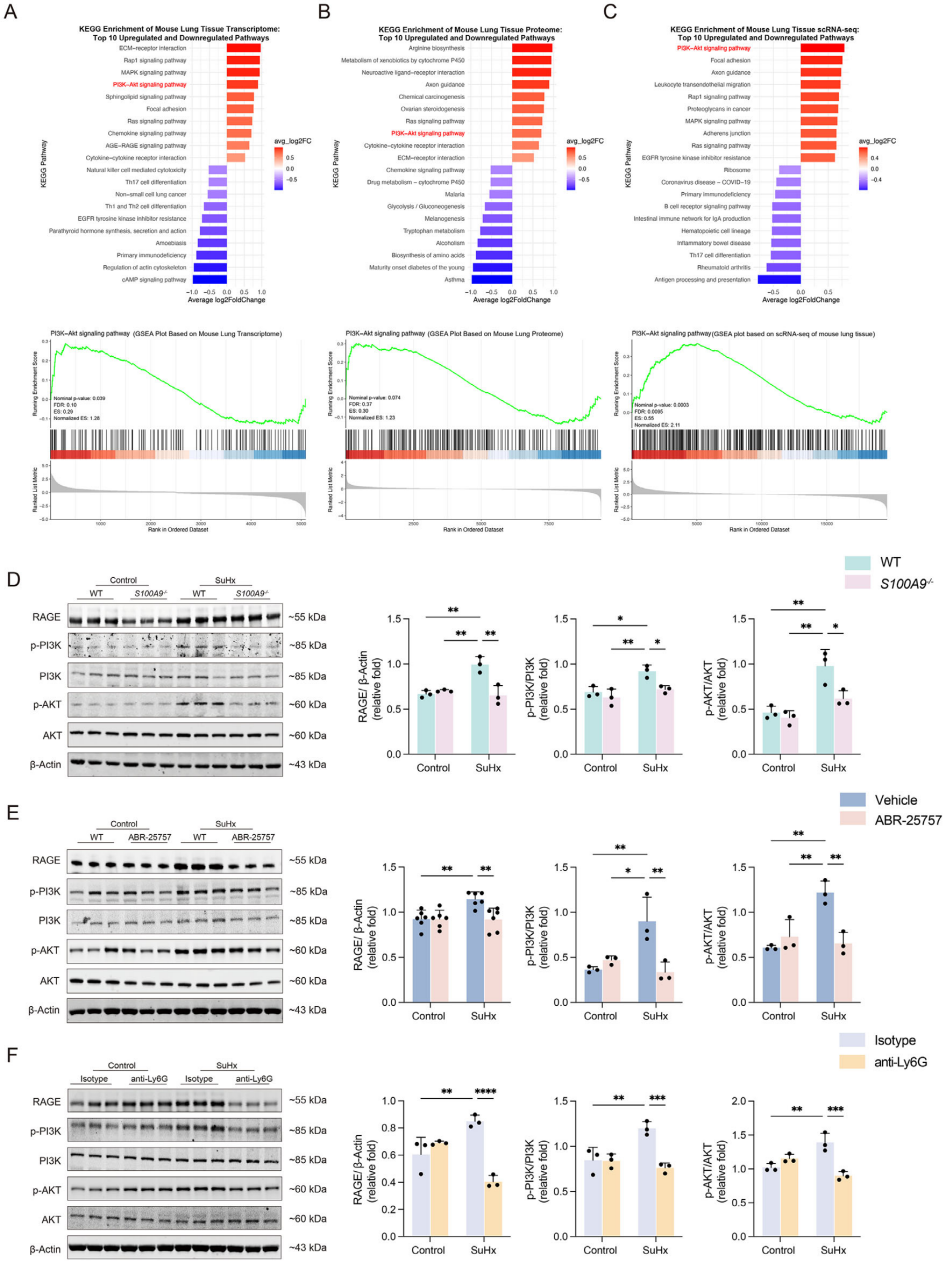
S100A9‐RAGE‐PI3K‐AKT pathway in murine model of SuHx‐induced PH. A) KEGG and GSEA pathway enrichment analysis of mouse lung tissue in transcriptome. B) KEGG and GSEA pathway enrichment analysis of mouse lung tissue in proteomics. C) KEGG and GSEA pathway enrichment analysis of mouse lung tissue in scRNA‐seq. D) Representative Western blot and quantification of protein expression in lung tissues of *S100A9^−^* ^/^ *^−^* mice, focusing on the RAGE/PI3K/AKT pathway (n = 3 per group). E) Representative Western blot and quantification of protein expression in lung tissues of mice treated with ABR‐25757, focusing on the RAGE/PI3K/AKT pathway (n = 3‐6 per group). F) Representative Western blot and quantification of protein expression in lung tissues of mice treated with anti‐Ly6G antibody, focusing on the RAGE/PI3K/AKT pathway (n = 3 per group). *p < 0.05, **p < 0.01, ***p < 0.001, ****p < 0.0001. Two‐way ANOVA was performed, followed by Bonferroni correction for six pairwise comparisons among the four experimental groups.

We next explored whether the expression level or presence of S100A9 affects the overall expression of the RAGE‐PI3K‐AKT pathways in vivo. We analyzed relevant protein levels in the lung tissues of mice with SuHx‐induced PH. To achieve these, the transcriptomic, proteomic, and single‐cell transcriptomic analyses were performed. KEGG and GSEA enrichment results showed that there were significant changes in PI3K‐AKT‐related pathways in mice with SuHx‐induced PH compared to those of normal controls across all three omics datasets (**Figure** **7**A–C).

Next, we analyzed protein levels of the RAGE‐PI3K‐AKT pathways in three SuHx‐induced PH animal models, including *S100A9* ^−/−^ mice, mice treated with ABR‐25757, and those of neutrophil depletion with anti‐Ly6G antibody, using Western blot (Figure 7D–F). The results indicated that either S100A9 knockout, reduced expression, or inhibition of its binding to the RAGE receptor all significantly suppressed SuHx‐induced activation of the RAGE‐PI3K‐AKT pathways. These findings suggest that S100A9 plays a crucial role in activating such pathway in SuHx‐induced PH.

## 3 Discussion

**Figure 8:**
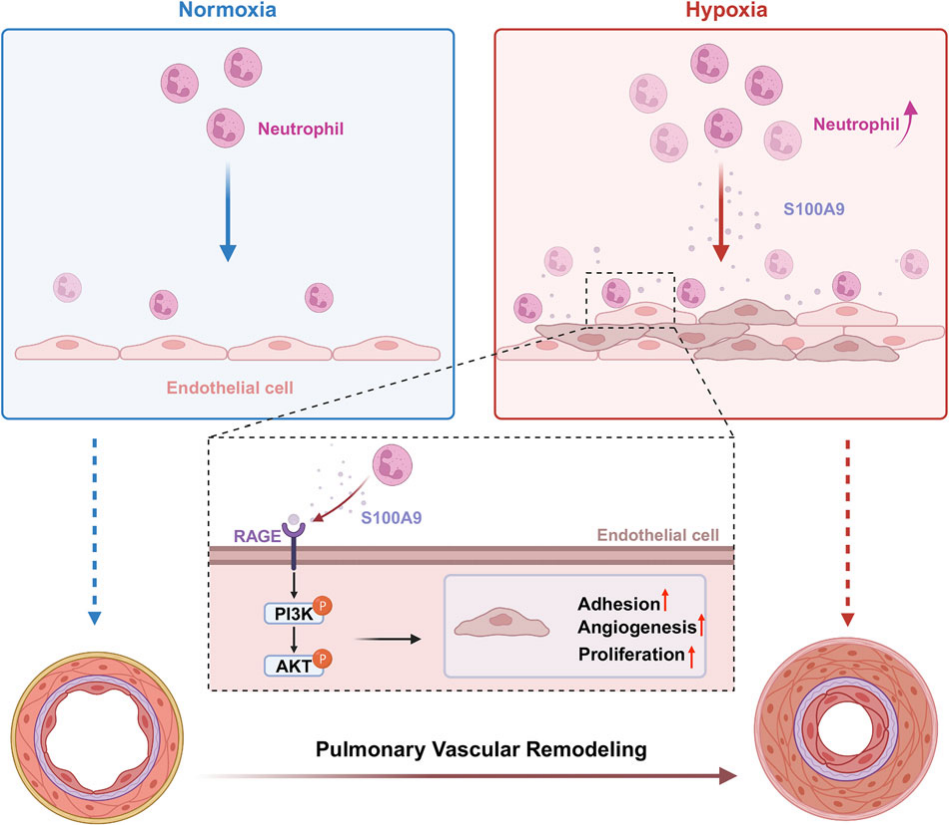
In the pulmonary vasculature, hypoxia not only increases numbers of neutrophils but also enhances the ability of these cells to adhere to ECs. These neutrophils release S100A9, which binds to RAGE receptors on ECs, and activates the RAGE‐PI3K‐AKT signaling pathway. This process further induces endothelial cell proliferation and vascular remodeling, contributing to the progression of PH. Thus, it is reasonably to speculate for that targeting S100A9 might be beneficial for patients with PH.

To the best of our knowledge, there are lack of studies regarding the role of neutrophils‐derived S100A9 in the development of SuHx‐induced PH and the potential underlying mechanisms. We first demonstrated that the expression of S100A9 increased during the development of SuHx‐induced PH, which was primarily from the increased neutrophils. Notably, both the depletion of neutrophils and the absence of S100A9 significantly attenuated symptoms of mice with SuHx‐induced PH. Furthermore, our results showed that neutrophils exposed to hypoxia impaired endothelial function, an effect that was abolished by inhibiting S100A9. Data of our mechanistic experiments indicated that S100A9 mediated endothelial dysfunction by activating the RAGE/PI3K/AKT pathways, and its upregulation enhanced neutrophil adhesion to ECs, thereby promoting the progression of SuHx‐induced PH. Therefore, S100A9 likely represents a promising anti‐inflammation therapeutic target for reversing pulmonary vascular remodeling (**Figure** **8**).

Currently, most clinical treatments for PH are vasoconstriction inhibitors, targeting the classical pathways of PH pathogenesis, such as endothelin‐1 (ET‐1),^[^ ^27^ ^]^ prostacyclin,^[^ ^28^ ^]^ and nitric oxide pathways.^[^ ^29^ ^]^ However, these approaches mainly focus to inhibit pulmonary vasoconstriction and reduce pulmonary vascular resistance but less fundamentally to address vascular remodeling. As the role of inflammation in pulmonary vascular dysfunction has become increasingly evident, immunotherapy has shown great promise for PH treatment.^[^ ^5^ ^]^ Over recent years, multiple strategies targeting immune cells, inflammatory cytokines, and related signaling pathways have been developed to effectively treat PH.^[^ ^30^, ^31^, ^32^ ^]^

As known, the altered inflammatory environment surrounding pulmonary blood vessels mainly results from immune cell infiltration and the production and recruitment of inflammatory factors. Our previous and ongoing studies have also confirmed that some cytokines, such as IL‐17^[^ ^11^ ^]^ and IL‐33,^[^ ^13^ ^]^ as well as immune cells, including T lymphocytes^[^ ^12^ ^]^ and macrophages, are potential immune factors contributing to pulmonary vascular remodeling in PH. Furthermore, numerous studies have demonstrated elevated levels of circulating immune cells in PH patients and animal models, with a significant increase in neutrophil counts,^[^ ^33^, ^34^ ^]^ however, the specific role of neutrophils in PH has yet to be fully elucidated. First, we observed an increased number of neutrophils in the peripheral blood and the lung tissues of mice with SuHx‐induced PH, as well as these cells aggregated around the blood vessels within the lung tissues of patients with PH. These observations are consistent with clinical findings showing an inverse relationship between neutrophil ratio and PH patient prognosis.^[^ ^35^ ^]^

Recent findings have revealed that ECs participate in a variety of biological processes. As the outermost layer of the vascular intima, ECs are the first to respond to stimuli and subsequently transmit activation signals to the outer layers, including smooth muscle cells, collectively mediating the formation of pulmonary vascular remodeling.^[^ ^7^ ^]^ Second, in vitro experiments also confirmed that, following hypoxia, neutrophils exhibited functional impairment, which in turn induces endothelial cell dysfunction. Therefore, it is evident that endothelial dysfunction may serve as one of the critical initiating factors in pulmonary vascular remodeling. These suggest that neutrophils likely play an important role in the progression of PH, either through promoting an inflammatory phenotype or directly contributing to vascular impairment, or both.

Thirdly, we found that there was the increase of S100A9 in the lung tissue of COPD‐PH patients. Such increased S100A9 was also confirmed in the lung tissues of numerous established PH models, including the MCT‐induced model and the chronic hypoxia and hypoxia combined with Sugen‐induced animal models. Furthermore, neutrophils are the major cellular source for S100A9 in clinical samples and our SuHx‐induced models. In recent years, the potential role of S100A9 in PH has received increasing attention. Taz et al. reported, through comparative genomic analyses, that SARS‐CoV‐2 infection upregulates the expression of the inflammation‐related protein S100A9, thereby exacerbating the inflammatory response and vascular injury, ultimately worsening the condition of PH patients. These findings suggest that S100A9 may serve as a potential therapeutic target in PH, and that inhibiting its expression or activity may alleviate SARS‐CoV‐2 infection‐associated PH deterioration.^[^ ^36^ ^]^ However, the precise molecular mechanisms of S100A9 in PH, as well as its predominant cellular source, remain incompletely understood. Our data suggest that neutrophil‐derived S100A9 might be a critical factor in the pathogenesis of PH, at least in mice with the SuHx‐induced PH. At the molecular level, S100A8 and S100A9, as key members of the S100 protein family, are typically co‐expressed in neutrophils and monocytes in the form of a heterodimer (S100A8/A9, also known as calprotectin).^[^ ^37^ ^]^ As damage‐associated molecular patterns (DAMPs), they are capable of amplifying immune responses.^[^ ^15^, ^22^ ^]^ For instance, Wu et al. demonstrated that S100A8/A9 released from CD11b⁺Gr1⁺ neutrophils activate cardiac fibroblasts, thereby promoting angiotensin II‐induced cardiac inflammation and injury.^[^ ^38^ ^]^ In our experiments, we also found that exogenous administration of the S100A8/A9 heterodimer similarly enhances endothelial cell functions, including proliferation, angiogenesis, and cell adhesion, with S100A9 likely playing a key role in these processes (Figure S6A,B, Supporting Information). Obviously, understanding the similarities and differences in the mechanisms of action between S100A9 alone and the S100A8/A9 heterodimer, as well as their interrelationship, will be one of the key focuses of any future relevant studies. Although both S100A8 and S100A9 are involved in the regulation of inflammation, their temporal roles and functional intensities might differ in the phases of inflammation, i.e., S100A8 primarily acts in the early phase of inflammation, whereas S100A9 is more responsible for sustaining and amplifying the inflammatory response.^[^ ^39^ ^]^

Finally, we demonstrated that targeting S100A9 or neutrophils either reversed S100A9‐induced changes in ECs in vitro or in vivo attenuated symptoms of mice with PH. While the S100A8/A9 complex is known to be an effective ligand for receptors such as RAGE and TLR4, the monomeric form of S100A9 has a significantly higher affinity for these receptors compared to the heterodimer.^[^ ^40^ ^]^ It has been reported that through interactions with RAGE or TLR4, S100A9 induces neutrophil migration and adhesion to ECs,^[^ ^41^ ^]^ as well as promotes the formation of a large number of neutrophil extracellular traps (NETs).^[^ ^42^ ^]^ On the other hand, the S100A9‐RAGE axis also induces endothelial dysfunction via F‐actin, ZO‐1, and occluding.^[^ ^43^ ^]^ Significantly, the PI3K‐AKT signaling pathway plays a crucial role in regulating these pathological processes mediated by S100A9. Tang et al. demonstrated that the deletion of AKT1 conferred protection against the development and progression of chronic hypoxic pulmonary hypertension, and played a dominant role in pulmonary vascular remodeling.^[^ ^44^ ^]^ Specifically, AKT1 might be involved in glycolytic shift and metabolic reprogramming, as well as endothelial dysfunction mediated by various factors including PHD2 and AIP1.^[^ ^45^, ^46^ ^]^ In the present study, we mainly focused on the activation of the PI3K/AKT pathway by S100A9 via its receptor RAGE, and its role in modulating endothelial cell function. However, PI3K/AKT may not be the only downstream signaling pathways involved in S100A9‐RAGE‐mediated endothelial dysfunction. Previous studies have shown that RAGE, as a multi‐ligand receptor, can interact with various signaling molecules and activate multiple downstream pathways, including MAPK, NF‐κB, and JAK/STAT, which are implicated in inflammation, apoptosis, and barrier regulation.^[^ ^47^, ^48^ ^]^ Therefore, it is reasonable to speculate that S100A9‐RAGE also acts through these non‐PI3K/AKT pathways to form a broader signaling network that co‐regulates endothelial cell proliferation, migration, and adhesion. In future studies, it is worth to further explore whether S100A9‐RAGE interacts with other signaling molecules using molecular intervention approaches, such as specific inhibitors or RAGE mutant constructs, to comprehensively elucidate the mechanisms underlying endothelial dysfunction. Consistent with these, our data showed that neutrophil‐derived S100A9 causes endothelial dysfunction through RAGE/PI3K/AKT pathways. Notably, both co‐culture of ECs with neutrophils and/or with exogenous rhS100A9 activated RAGE expression. However, exogenous rhS100A9 did not significantly activate TLR4 (Figure S4, Supporting Information), another receptor of S100A9, suggesting that RAGE may play a more dominant role. Moreover, these pathological changes were alleviated following treatment with the S100A9 inhibitor Paquinimod.

Taken together, S100A9 appears to be a crucial inflammatory molecule during the process of pulmonary vascular remodeling, suggesting that S100A9 likely a potential molecule for therapeutic target in PH. In the development of drugs targeting S100A9, Paquinimod, which blocked its interaction with RAGE and TLR4, has already reached Phase II clinical trials for the treatment of systemic sclerosis, demonstrating promising efficacy and safety.^[^ ^24^ ^]^ Our study further sheds likely light on the development of novel treatment strategy for PH. However, the present study also has certain limitations. One notable limitation is the lack of sex‐specific analyses, which is particularly important given that estrogen‐mediated autophagy attenuation may contribute to improved survival and better right ventricular function in female PH patients.^[^ ^49^ ^]^ Second, while we have demonstrated that neutrophils mediate endothelial dysfunction through the secretion of S100A9, whether this process further drives abnormal proliferation and dysfunction of smooth muscle cells remains unclear. Finally, although both the S100A9 knockout model and the pharmacological inhibition model affect S100A9 expression and function at a systemic level, however, due to technical limitations and the intrinsic characteristics of neutrophils, we were unable to further investigate the specific effects of targeting S100A9 in neutrophils. Investigating this mechanism will be a key focus of our future research.

In conclusion, our data show that targeting the neutrophil‐S100A9‐RAGE‐endothelial cell axis could represent a novel therapeutic approach for managing PH, potentially improving outcomes for patients suffering from the disease. In addition, considering the crucial role of neutrophils in pulmonary vascular remodeling, our findings also highlight the importance of controlling and preventing infections as a key strategy in clinical practice to hinder the progression of PH.

## 4 Experimental Section

### Ethical Statements

The present study was approved by the Ethics Committee of Capital Medical University and conducted in accordance with the principles of the Declaration of Helsinki. Informed consent was obtained from all patients or their relatives involved in lung tissue collection.

All animal experiments were approved by the Animal Review Committee of Capital Medical University (Approval Numbers: AEEI‐2020‐100, AEEI‐2021‐156) and conducted in strict accordance with the Regulations for the Administration of Affairs Concerning Experimental Animals. All procedures also conformed to the guidelines of Directive 2010/63/EU of the European Parliament on the protection of animals used for scientific purposes. The study adhered to the 3R principles (Replacement, Reduction, and Refinement) to ensure the ethical and scientific integrity of animal research.

### Human Lung Samples

Lung tissues were, either via lung transplantation or lobectomy, collected from donors, patients with chronic obstructive pulmonary disease (COPD) only, and those with COPD‐PH, fixed in 10% formaldehyde, and then embedded in paraffin. The patients’ clinical data can be seen in our previous published paper^[^ ^50^ ^]^ and Table S1 (Supporting Information).

### Animal Models

C57BL/6 mice and Sprague Dawley rats were purchased from Beijing Vital River Laboratory Animal Technology Co., Ltd. Global S100A9 knockout (KO) mice *(S100A9^−/−^* mice, C57BL/6 background, *KOCMP‐20202‐S100A9‐B6N‐VA)* were kindly provided by Professor Huihua Li from Beijing Chao‐Yang hospital.^[^ ^51^, ^52^ ^]^ Animals were randomly assigned to various treatment groups for each experiment. Mice and rats were maintained on a 12 h light‐dark cycle with a regular unrestricted diet. To establish a Sugen‐hypoxia‐induced PH mouse model (SuHx), 8‐10‐week‐old male mice received weekly subcutaneous injections of SU5416 (20 mg·kg^−1^/per does; Selleck, catalog no. S2845, TX) for 4 weeks and were housed in an airtight plexiglass chamber with 10% O_2_.^[^ ^53^ ^]^ To establish a chronic‐hypoxia‐induced PH mouse model, 8‐10‐week‐old male mice were housed in an airtight plexiglass chamber with 10% O_2_ for 4 weeks. To generate monocrotaline (MCT)‐induced PH, male Sprague Dawley rats (≈200–250 g) received a single intraperitoneal injection of MCT (60 mg·kg^−1^/per dose; MCE, catalog no. HY‐N075, NJ) followed by a 4‐week observation period. For the murine model with neutrophil depletion, isotype IgG (Selleck, catalog no. A2123, TX) or anti‐Ly6G‐antibody (Selleck, A2158, TX, 200 µg per dose) was injected intraperitoneally 24 h prior to the assay, followed by injections alternate days. In the Paquinimod group, the Paquinimod (Selleck, catalog no. S9963, TX) was given 2 days prior to the start of the assay (10 mg·kg^−1^/per dose), and the next consecutive 4 weeks, while their control group was given equal amount of 1 × PBS injection.

All animals were anesthetized with isoflurane (3% for induction, 1.5% for maintenance), administered via inhalation using a calibrated vaporizer. For terminal procedures, an overdose of sodium pentobarbital (150 mg kg^−1^) was administered by intraperitoneal injection. All efforts were made to minimize the suffering of the animals.

### ScRNA‐Seq and Analysis

To obtain single‐cell RNA‐seq (scRNA‐seq) data, lung tissues of the experimental mice were harvested and analyzed using 10x Genomics Chromium single‐cell 3’v2 and a V(D)J kit (10×Genomics) according to the manufacturer's instructions. Libraries were sequenced at 150 paired‐end cycles using the HiSeq3000 system (Illumina). The differentially expressed genes (DEGs) of each cluster were identified using “FindAllMarkers” function, and the clusters were annotated based on classic marker genes. Kyoto Encyclopaedia of Genes and Genomes (KEGG) analysis was performed by “clusterProfiler” R package. “CellChat” package was used to evaluate the interactions between cells. Detailed information regarding the R packages and analytical procedures is provided in the Table S3 (Supporting Information).

Cell–cell communication was inferred using the R package CellChat (v1.6.1). The Seurat object containing the annotated scRNA‐seq data was converted to a CellChat object. Genes were filtered to retain only those expressed in more than 10% of cells within each cell group. The built‐in database CellChatDB.mouse was used to identify known ligand‐receptor pairs. Overexpressed genes and interactions were identified using default thresholds, followed by the computation of communication probabilities based on a mass action model. Cell‐cell communication networks were visualized using heatmaps, circle plots.

### Transcriptomic and Proteomic Data Analysis and Pathway Enrichment

Lung tissues were collected from the experimental mice, total RNAs were extracted for transcriptomic analysis, while proteins were isolated separately for proteomic analysis. Transcriptomic analysis was performed using RNA sequencing, while proteomic analysis was conducted through mass spectrometry for protein identification and quantification.

### Hemodynamic and Ventricular Weight Measurements

The right ventricular systolic pressure (RVSP) was measured through closed‐chest insertion of the experimental mice. Briefly, after anesthesia, a 22‐gauge needle connected to a pressure transducer interfaced with a PowerLab system (AD Instruments, Australia) was inserted into the RVs of anesthetized animals through a xiphocostal angle approach, and the waveform was used to confirm the position of the needle. Prior to RVSP measurement, the RV wall thickness (RVWT), tricuspid annular plane systolic excursion (TAPSE), and pulmonary artery acceleration time (PAAT) were measured using a Vevo 2100 Imaging system (FUJIFILM VisualSonics, lnc., SC). The results were calculated using Visual Sonics Vevo 2100 analysis software (v.1.6) with a cardiac measurement package, based on the average of three cardiac cycles.

### Histological Analyses

Animals were anesthetized with sodium pentobarbital, sacrificed after hemodynamic measurements, and perfused with chilled saline. Right lung and heart tissues were fixed with 10% formaldehyde at 25 cm H_2_O pressure and processed for histological and immunostaining analyses. For H&E staining, 4‐µm lung sections were analyzed for medial thickness (MT%) and medial area of pulmonary vessels (≤100 µm external diameter) using a Nikon microscope and image analysis software. For IHC staining, slides underwent antigen retrieval (citric acid buffer, pH 6.0), blocking, and incubation with primary antibodies against neutrophil elastase (ab68672, 1:2500, Abcam) and S100A9 (#73425, 1:200, CST), followed by HRP‐conjugated secondary antibodies and DAB development. For IF staining, slides were incubated with primary antibodies against CD31 (ab182981, 1:200, Abcam), Ly6G (ab25377, 1:200, Abcam), MPO (ab20257, 1:200, Abcam), α‐SMA (#BM0002, 1:200, Boster), and S100A9 (#73425, 1:200, CST), followed by Alexa Fluor‐conjugated secondary antibodies and DAPI nuclear staining. For WGA staining, slides were treated with 5 mM WGA (W11262, Invitrogen) and counterstained with DAPI. Quantification of positive staining was performed on nine randomly selected images per mouse using Image‐J, with positive cell counts normalized to field area (mm^2^).

### Flow Cytometry

Neutrophil from blood and lung tissues of mice were analyzed by flow cytometry. In brief, single‐cell suspensions of mouse lung tissue were prepared by repeated rounds of enzymatic digestion and trituration with collagenase type I (Roche, Switzerland), neutral protease and DNase I (Sigma, catalog no. 10104159001, CA). Primary antibodies of APC‐Cy^TM^7‐CD45 (BD, catalog no. 557659, NJ), PE‐Ly6G (Invitrogen, catalog no. 12‐5931‐82, CA) and FITC‐CD11b (Invitrogen, catalog no. 11‐0112‐82, CA) were used at a 1:100 dilution. The results were collected by BD FACSymphony A5, and analyzed with FlowJo 10.8.1 software. More than 10,000 events were collected. Live cells were selected in FSC_SSC dot plot. The single cells were chosen in FSC‐A_FSC‐H dot plot. Events in the single‐cell gate were analyzed. After sorting the live cells and single cells, at least 5,000 events were ready for further analysis.

### Western Blot Analyses

Protein extracts were prepared by lung tissue in ice‐cold RIPA buffer supplemented with protease and phosphatase inhibitors (Roche, catalog no. 04693116001 and 04906837001, Switzerland), and protein concentration was determined using the BCA protein assay kit (Thermo Fisher Scientific, catalog no. A55860, CA). Equal amounts of protein 20 µg were resolved by SDS‐PAGE and transferred to NC membranes (Millipore, catalog no. HATF00010, MA). Membranes were blocked with a rapid blocking buffer (Epizyme Biotech, catalog no. PS108P, China) for 10–15 min at room temperature and then incubated with primary antibody RAGE (Abcam, catalog no. ab216329, 1:1,000 dilution, CA), S100A9 (CST, catalog no. 73425, 1:1,000 dilution, CA), PI3K (CST, catalog no. #4249, 1:1,000 dilution, CA), Phospho‐PI3K (CST, catalog no. #4228, 1:500 dilution), Akt (CST, catalog no. #9272, 1:1,000 dilution), and Phospho‐Akt (CST, catalog no. #9271, 1:500 dilution), membranes were incubated with fluorophore‐conjugated secondary antibodies (LI‐COR, catalog no. 926–32211 and 926–68020, I:5,000 dilution, CA) for 1 h at room temperature. Protein bands were detected using an Odyssey infrared imaging system (LI‐COR Biosciences, CA). Band intensities were quantified using Image Studio software (LI‐COR Biosciences), and relative protein levels were normalized to the loading control.

### ELISA Detection of Serum S100A9

Concentrations of human serum S100A9 were measured using an ELISA kit (Biolegend, catalog no. 445307, CA) according to the manufacturer's instructions.

### Cell Culture and Preparation

Human pulmonary artery endothelial cells (hPAECs) from normal human subjects (ScienCell Research Laboratories, catalog no. 3100, CA) were cultured in culture medium (EC Medium, ECM, ScienCell Research Laboratories, catalog no. 1001, CA), supplemented with Penicillin/Streptomycin (P/S), growth supplements and 5% (v/v) fetal bovine serum (FBS) and maintained at 37°C in a humidified normoxia condition (21% O_2_, 5% CO_2_, 74% N_2_) or hypoxia condition (3% O_2_, 5% CO_2_, 92% N_2_). Cells were passaged (passages 3–5) after reaching 80–90% confluence, detached with 0.05% trypsin, 0.04% EDTA (Sigma‐Aldrich, catalog no. EDS‐100G, Germany) in phosphate‐buffered saline (PBS).

Human neutrophils were collected from fresh peripheral blood of healthy volunteers using EDTA or heparin as an anticoagulant to prevent clotting, and separated using Histopaque‐1119 (Sigma‐Aldrich, catalog no. 11191, Germany) and Histopaque‐1077 (Sigma‐Aldrich, catalog no. 10771, Germany). After separation, flow cytometry is used for validation.

### Tubule Formation and Adhesion Assay of hPAECs

To detect the tube formation ability of hPAECs, a tube formation assay was performed. BD Matrigel (BD Biosciences, catalog no. 356234, NJ) was distributed in a 24‐well plate (200 µL/well) on ice and allowed to solidify at 37 °C for at least 30 min. After the Matrigel solidified, hPAECs (1 × 10^5^ per well) were gently added to each of the triplicate wells. Tube formations were quantitatively measured by calculating the number of tube‐like structures using NIS‐Elements Basic Research‐Microscope Imaging Software (Nikon, Japan). Tracks of hPAECs organized into networks of cellular cords were counted in 6 random fields. Additionally, for the cell adhesion assay, hPAECs were trypsinized and seeded on top of fibronectin (30 µL of 20 µg mL^−1^; Millipore, catalog no. FC010, MA)‐coated 96‐well plates (1×10^4^ per well in 5% FBS and growth factor–free ECM) and incubated for 30 min at 37 °C. After washing with PBS, the adhered cells were photographed (4 pictures per well, 5 wells per group), and the data were analyzed using ImageJ software.

### Neutrophil Adhesion Assay

Neutrophils were labeled with PKH26 according to the manufacturer's instructions (Sigma‐Aldrich, PCLDIL, Germany). After that, hPAECs were then co‐cultured with PKH26‐labeled neutrophils at a concentration of 2 × 10^5^ mL^−1^ for 1 h. The culture medium and unattached cells were removed by washing twice with PBS. Slides were mounted and images were captured to record the PKH26‐labeled neutrophils.^[^ ^54^ ^]^

### Statistical Analyses

All statistical analyses were performed using GraphPad Prism version 10.2.3. Data are presented as means ± standard deviation (SD). For comparisons between two groups, an unpaired t‐test was applied. For comparisons among three, four or six groups, one‐way analysis of variance (ANOVA) was performed, followed by Bonferroni post hoc correction applied to three, six or fifteen pairwise comparisons. For comparisons among four experimental groups, two‐way ANOVA was performed, followed by Bonferroni post hoc correction applied to six pairwise comparisons. A P value less than 0.05 was considered statistically significant. Randomization and blinded analyses were applied whenever possible.

### Data Availability

Sequencing data generated in the present study have been deposited at the Genome Sequence Archive (https://ngdc.cncb.ac.cn/gsa) and are publicly available at the date of publication. The accession nos. of scRNA‐seq, proteomic and RNA‐seq data are CRA020769, OMIX008029 and CRA020767, respectively.

## Conflict of Interest

The authors declare no conflict of interest.

## Author Contributions

Y.G. performed investigation, methodology, visualization, and wrote‐original draft. Z.G. performed formal analysis, investigation, and methodology. R.Z. performed software, formal analysis, data curation, and visualization. H.L. performed software, validation, and visualization. M.Z. performed formal analysis, and data curation. L.L., Z.A., and Y.S. performed software. Y.C. performed supervision. Y.J. performed conceptualization. L.W. performed conceptualization, wrote, reviewed, and edited. Y.S. performed conceptualization, supervision, project administration, funding acquisition, writote, reviewed, and edited. J.L. performed conceptualization, supervision, project administration, funding acquisition. W.W. performed conceptualization, supervision, project administration, and funding acquisition.

## Supporting information



Supporting Information

## Data Availability

The data that support the findings of this study are available from the corresponding author upon reasonable request.
