# Supplementary material for: Neutrophil–Endothelium Interaction Mediated by S100A9 Promotes Pulmonary Vascular Remodeling During Pulmonary Hypertension
Source: Adv Sci (Weinh). 2025 Jun 10;12(31):e04397. doi: 10.1002/advs.202504397 (PMC12376707; doi:10.1002/advs.202504397)
Supplement: Supplementary file 1 — Supporting Information [file ADVS-12-e04397-s001.docx]

**Supplementary Information**

**Single-cell Transcriptomic Data Preprocessing and Downstream Analyses**

Original scRNA-seq datasets were initially imported into R (version 4.4.3) and analyzed using the Seurat package (version5.2.1). Raw gene expression matrices were converted into Seurat objects, and standard quality control filtering was performed by removing cells with fewer than 200 detected genes, more than 20% mitochondrial gene content, or extremely high gene counts (>5000). Normalization and variance stabilization were performed using the SCTransform (SCT) method, which mitigates technical noise while preserving biological variation. To correct for batch effects across the three datasets, the Harmony algorithm (version 1.2.3) was applied to the PCA embeddings generated from SCT-normalized data. The corrected embeddings were subsequently used for dimensionality reduction, clustering, and visualization via UMAP.

Cell clusters were annotated based on canonical marker gene expression. Differential gene expression analysis was performed using Seurat’s FindMarkers function, and downstream pathway enrichment analyses were conducted using clusterProfiler.

Differential abundance analysis was performed using MiloR (version 2.2.0). Neighborhood graphs were constructed and cells were assigned to local neighborhoods. A generalized linear mixed model (GLMM) was applied to detect differentially abundant neighborhoods between conditions, with an FDR < 0.1 used to define significance.

For functional enrichment analysis, DEGs were subjected to KEGG pathway enrichment analysis using the clusterProfiler package (version 4.14.6). In addition, Gene Set Enrichment Analysis (GSEA) was conducted to explore pathway activation in specific cell types under different conditions.

Table S1. Basic characteristics of patients and their comparison.

|  | **Control Subject**  **(n = 3)** | **COPD**  **(n = 3)** | **COPD-PH**  **(n = 3)** | ***P value** |
| --- | --- | --- | --- | --- |
| **Age at sampling (yr)** | 32.00±10.00 | 55.67±4.51 | 63.33±4.51 | 0.1059 |
| **Male:Female** | 3:0 | 3:0 | 2:1 | – |
| **Smoking (pack-year)** | N/A | 23.50±15.06 | 13.33±11.55 | 0.4059 |
| **sPAP (mmHg)** | N/A | normal | 48.33±15.63 | – |
| **Comorbid conditions** | | | | |
| **Hypertension** | 0 | 1 | 1 | – |
| **Tumor** | 0 | 1 | 0 | – |
| **Tuberculosis (past)** | 0 | 0 | 1 | – |
| **Depression** | 0 | 1 | 0 | – |

N/A, not available; PH, pulmonary hypertension; sPAP, systolic pulmonary artery pressure; *, difference between COPD and COPD-PH group (Unpaired t test). Data is shown as mean ± SD.

Table S2. Cluster cell and marker genes per cell type.

| Cluster | Cellt | Marker gene |
| --- | --- | --- |
| 0 | B | Cd19, Ms4a1 |
| 1 | Neutrophil | Fcgr3, Retnlg |
| 2 | Endothelial | Cldn5, Cdh5 |
| 3 | AM | Ctsd, Lpl, Plet1, Chil3 |
| 4 | T | Cd3d, Cd3e |
| 5 | cMonocyte | Ly6c2, Plac8 |
| 6 | NKT | Cd3d, Cd3e, Nkg7 |
| 7 | NK | Nkg7 |
| 8 | Fibroblast | Col1a1, Pdgfra, Col1a2, Bmp4 |
| 9 | ncMonocyte | Ly6c2-, Plac8+ |
| 10 | DC | Batf3, Irf4 |
| 11 | SMC | Des, Acta2, Tagln |
| 12 | IM | C1qb |
| 13 | proliferating AM | Pclaf, Mki67 |
| 14 | AT1 | Krt8, Hopx, Aqp5 |
| 15 | AT2 | Sftpa1, Muc1, Cxcl15, Sftpd |
| 16 | MAST | Ms4a2, Cpa3 |
| 17 | Megakaryocytes | Ppbp, Itga2b |

Table S3. R packages and their versions used in this study

All analyses were conducted in R (version 4.4.3).

| R package | Version |
| --- | --- |
| BiocManager | 1.30.25 |
| dplyr | 1.1.4 |
| Seurat | 5.2.1 |
| patchwork | 1.3.0 |
| ggplot2 | 3.5.2 |
| clustree | 0.5.1 |
| cluster | 2.1.8.1 |
| pheatmap | 1.0.12 |
| devtools | 2.5.4 |
| harmony | 1.2.3 |
| tidyverse | 2.0.0 |
| ggsci | 3.2.0 |
| rliger | 2.1.0 |
| clusterProfiler | 4.14.6 |
| msigdbr | 10.0.2 |
| org.Mm.eg.db | 3.20.0 |
| Cellchat | 1.6.1 |
| edgeR | 4.4.2 |
| openxlsx | 4.2.8 |
| miloR | 2.2.0 |

**Figure S1. Proportion changes in neutrophil numbers of lung tissues of mice with SuHx-induced PH and those of control mice.**

Flow cytometry gate strategy for neutrophils in lung tissues.

**Figure S2. Proportional changes in neutrophil numbers in the blood and lung tissues of SuHx-induced PH model mice treated with Ly6G antibody or isotype control.**

**A**, Flow cytometry gate strategy for neutrophils in blood. **B**, Flow cytometry gate strategy for neutrophils in lung tissues.


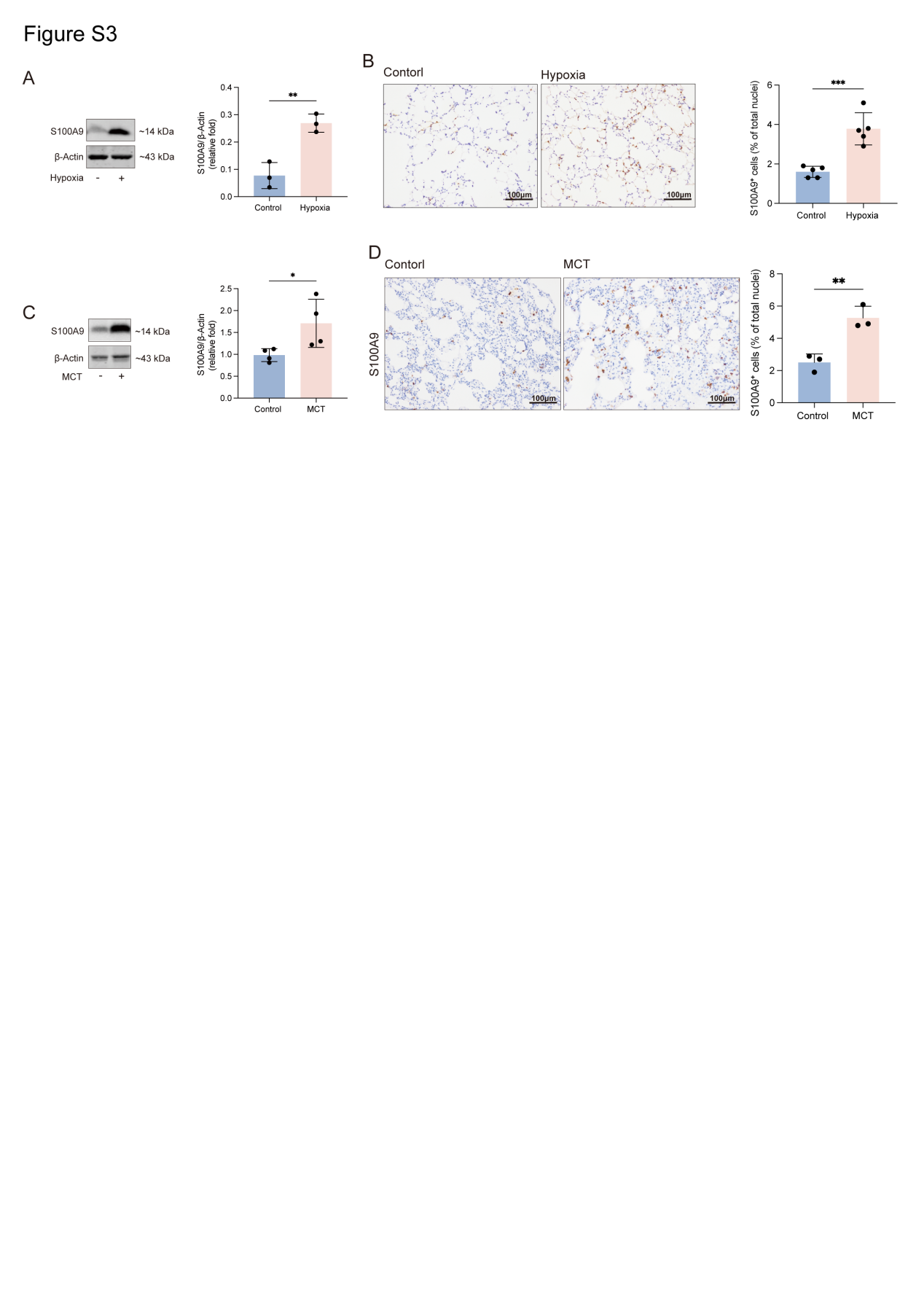


**Figure S3. S100A9 is upregulated in chronic hypoxia-induced PH mice and MCT-induced PAH rats.**

**A**, Representative Western blot and quantification of S100A9 expression in the lungs of mice with chronic hypoxia-induced PH (n=3 per group). **B**, IHC and quantity analysis of S100A9 in sections of lung tissues of mice with chronic hypoxia-induced PH (n=5 per group). **C**, Representative Western blot and quantification of S100A9 expression in the lungs of rats with MCT-induced PH (n=4 per group). **D**, IHC and quantity analysis of S100A9 in sections of lung tissues of mice with MCT-induced PH (n=3 per group). **p*<0.05, ***p*<0.01, *****p*<0.0001. Unpaired t-test was used.


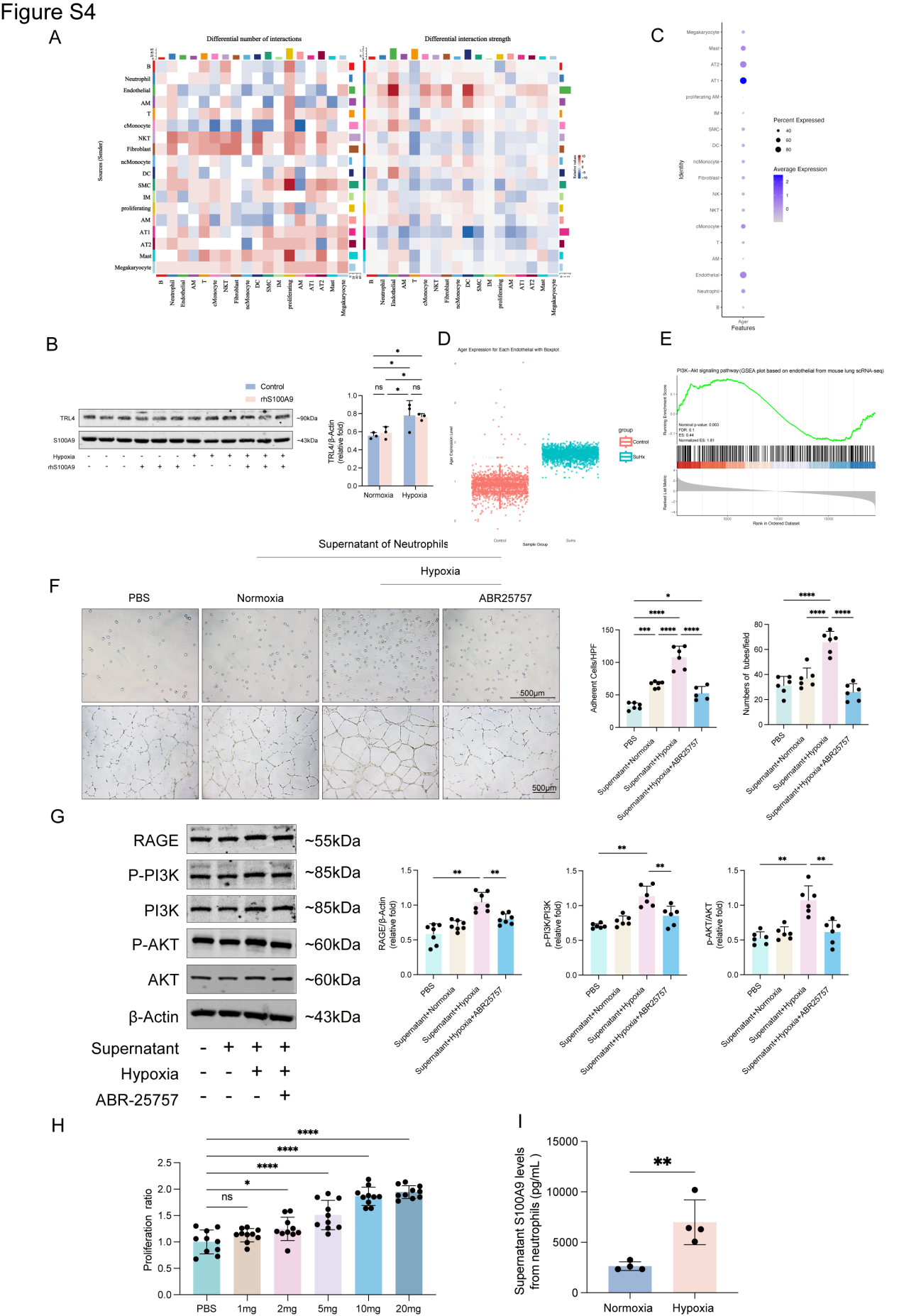


**Figure S4. S100A9-RAGE-PI3K/AKT axis mediates neutrophil-endothelial interactions and regulates endothelial dysfunction in pulmonary hypertension.**

**A**, Number and strength of cellular interactions were analyzed using ‘CellChat’, with results shown in a heatmap. **B,** Representative Western blot and quantification of changes in TLR4 of hPAECs induced by rhS100A9 under normoxic or hypoxic conditions (n=3 per group). **C,** Dot plot showing the expression level of Ager across different cell types. **D,** Boxplot showing the expression level of Ager in endothelial cells across different groups. **E,** GSEA pathway enrichment analysis of mouse lung endothelial cells. **F,** Representative images and quantification of adhesion and angiogenesis of hPAECs with supernatant of neutrophil under hypoxic or normoxic conditions, treated with or without ABR-25757 (n=5-6 per group). **G**, Representative Western blot and quantification of changes in RAGE/PI3K/AKT pathways of hPAECs cultured with supernatant of neutrophils under normoxic or hypoxic conditions, in the presence or the absence of ABR-25757 (n=6 per group). **H**, CCK8 analysis of hPAECs cell proliferation with different concentrations of rhS100A9 (n=10 per group). **I,** ELISA analysis of S100A9 in neutrophil supernatant (n=4 per group). ns *p*>0.05, **p*<0.05, ***p*<0.01, ****p*<0.001, *****p*<0.0001. Two-way ANOVA with Bonferroni correction was used for four-group comparisons (B). One-way ANOVA with Bonferroni post hoc was applied for six-group (F and G) and fifteen-group (H) comparisons. Unpaired t-test was used for two-group comparisons (I).


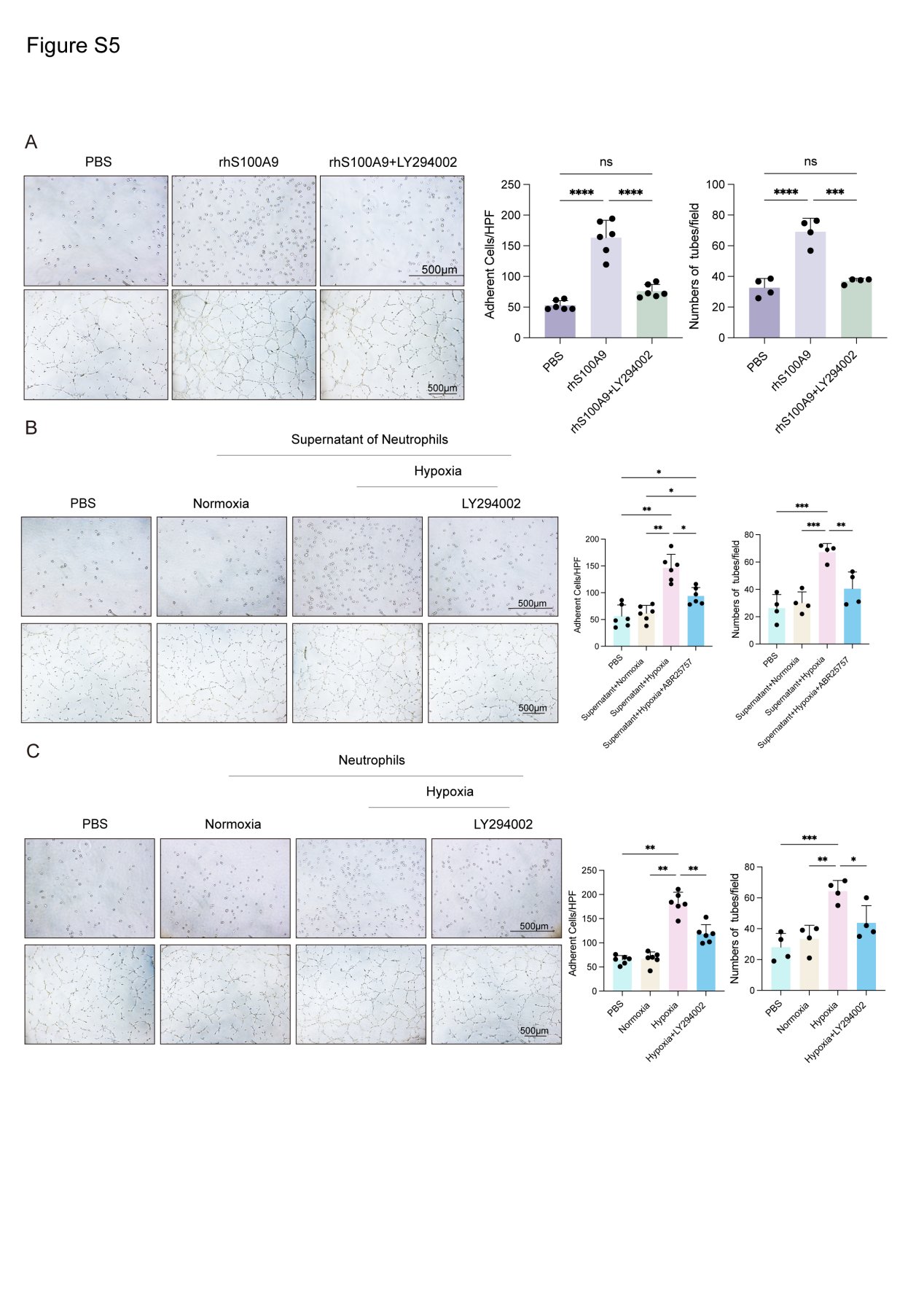


**Figure S5: PI3K inhibition reduces hPAEC adhesion and angiogenesis induced by S100A9 or neutrophil stimulation.**

**A,** Representative images and quantification of adhesion and angiogenesis of hPAECs evaluated after stimulation with rhS100A9 or treated with LY294002 (n=4-6 per group). **B,** Representative images and quantification of adhesion and angiogenesis of hPAECs with supernatant of neutrophil under hypoxic or normoxic conditions, treated with or without LY294002 (n=4-6 per group). **C,** Representative images of adhesion and angiogenesis of hPAESs co-cultured with neutrophil under hypoxic or normoxic conditions, in the presence or the absence of LY294002 (n=4-6 per group). One-way ANOVA was performed, followed by Bonferroni post hoc correction to account for multiple comparisons among the three groups (A) and the six groups (B and C).


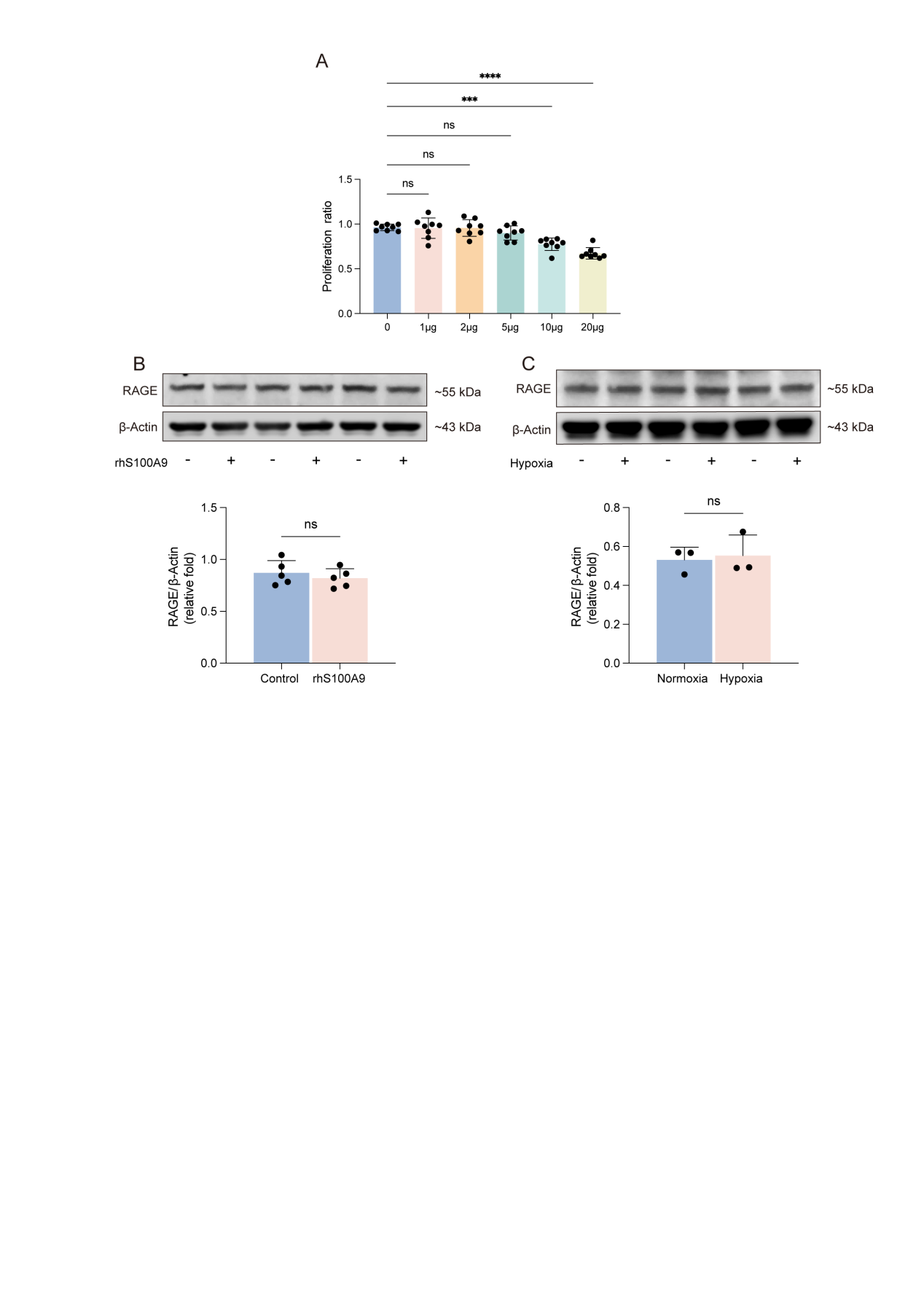


**Figure S6: Effects of S100A8/A9 and ABR-25757 on hPAEC function.**

**A,** CCK8 analysis of hPAECs cell proliferation with different concentrations of rhS100A8/A9 (n=9-10 per group). **B,** Representative images and quantification of adhesion and angiogenesis of hPAECs evaluated after stimulation with rhS100A8/A9 or treated with ABR-25757 (n=4-5 per group). ^ns^*p*>0.05, **p*<0.05, ***p*<0.01, ****p*<0.001, *****p*<0.0001. One-way ANOVA was performed, followed by Bonferroni post hoc correction to account for multiple comparisons among the fifteen groups (A) and the three groups (B).
